# Supplementary figures and images for: Precision RNAi using synthetic shRNAmir target sites
Source: eLife. 2023 Aug 8;12:RP84792. doi: 10.7554/eLife.84792 (PMC10409502; doi:10.7554/eLife.84792)

Precision RNAi using synthetic shRNAmir target sites

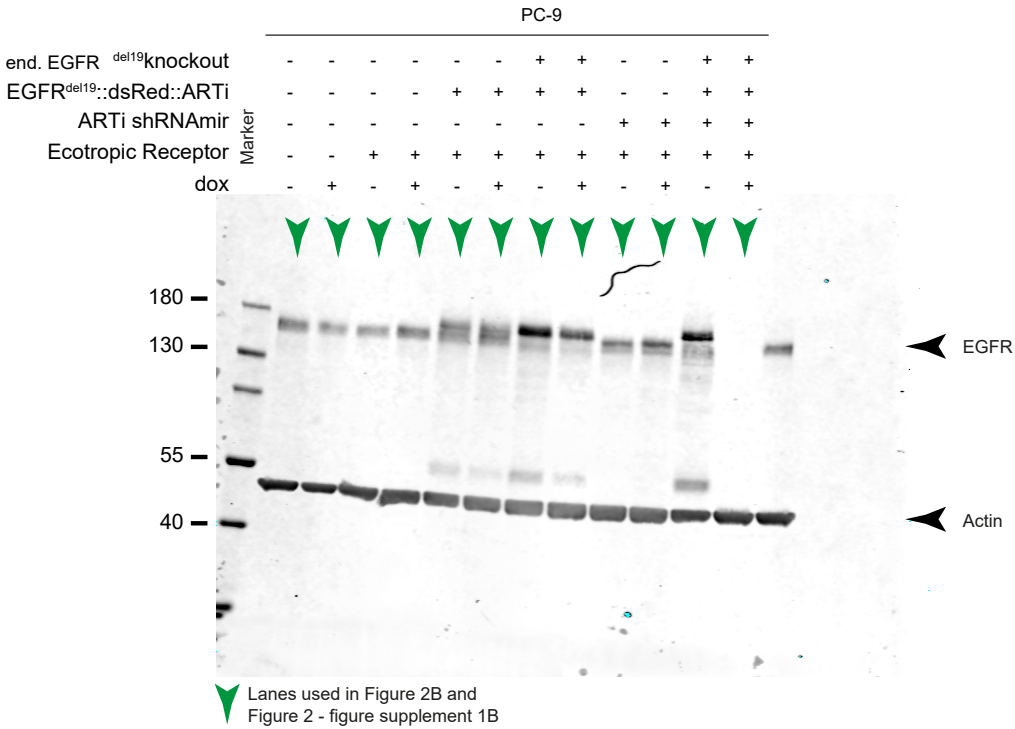

Supplement: Figure 2—source data 1. [file elife-84792-fig2-data1.zip › Figure2-source_data_1/Figure2_source_data1_annotated.pdf]

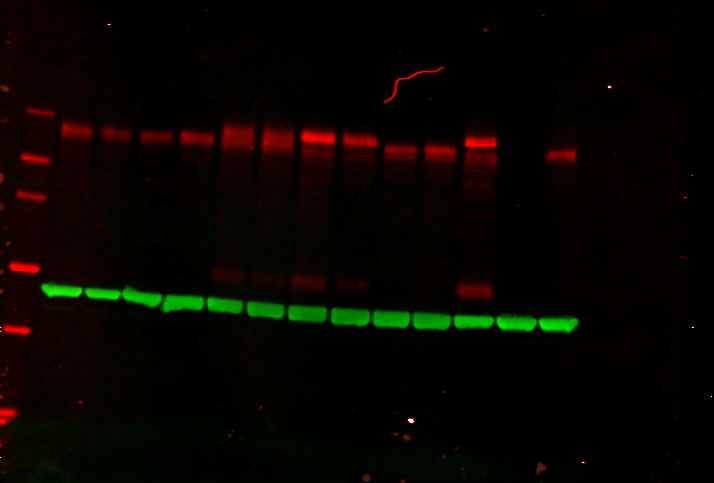

Supplement: Figure 2—source data 1. [file elife-84792-fig2-data1.zip › Figure2-source_data_1/Figure2-source_data1.tif]

Precision RNAi using synthetic shRNAmir target sites

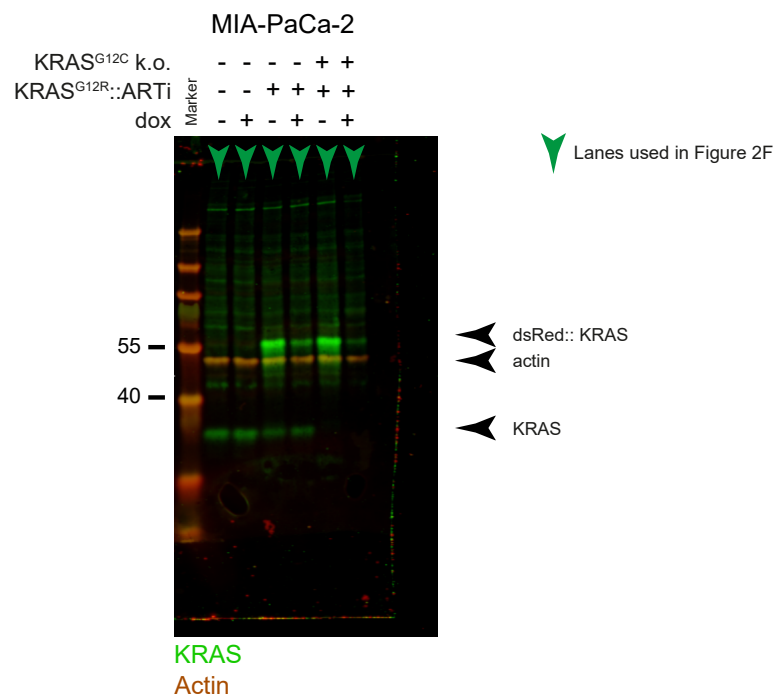

Supplement: Figure 2—source data 2. [file elife-84792-fig2-data2.zip › Figure2-source_data_2/Figure2_source_data2_annotated.pdf]

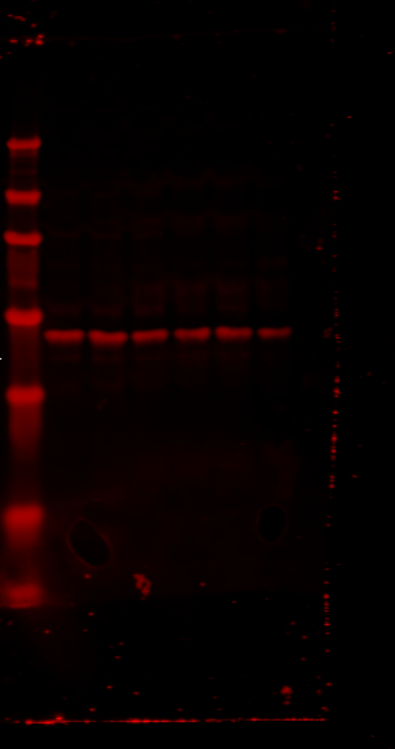

Supplement: Figure 2—source data 2. [file elife-84792-fig2-data2.zip › Figure2-source_data_2/Figure2-source_data2_actin.tif]

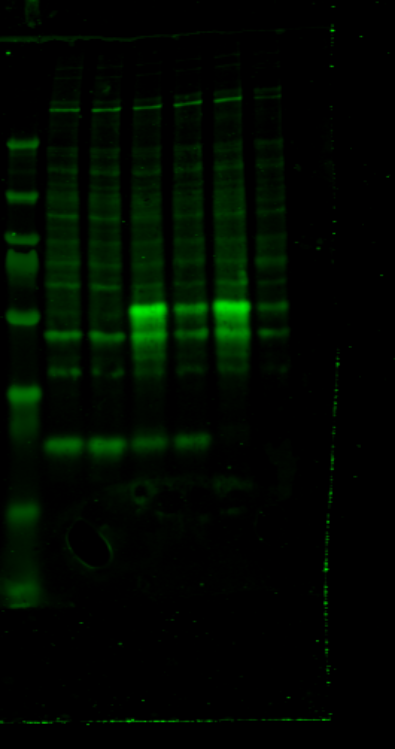

Supplement: Figure 2—source data 2. [file elife-84792-fig2-data2.zip › Figure2-source_data_2/Figure2-source_data2_KRAS.tif]

Precision RNAi using synthetic shRNAmir target sites

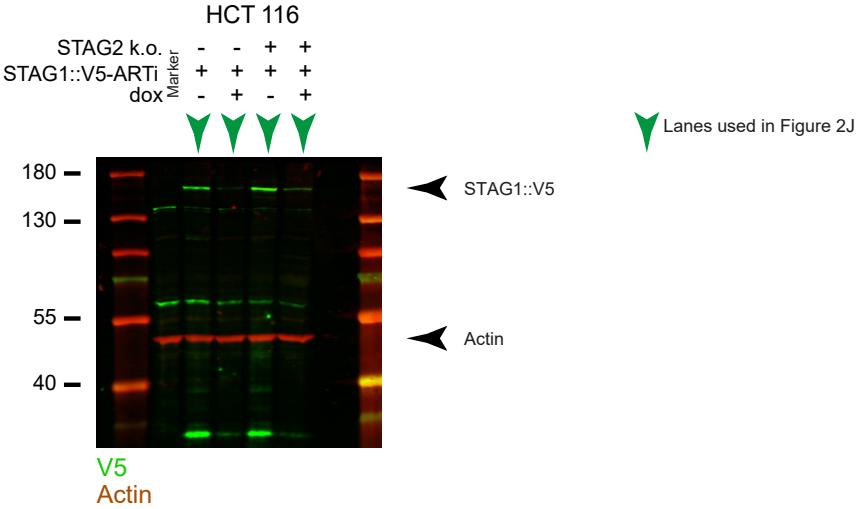

Supplement: Figure 2—source data 3. [file elife-84792-fig2-data3.zip › Figure2-source_data_3/Figure2_source_data3_annotated.pdf]

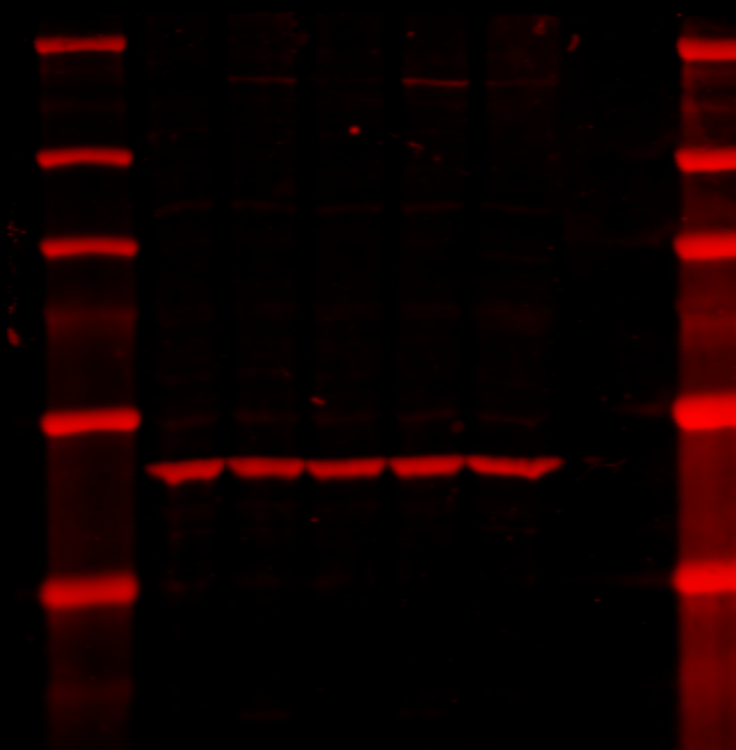

Supplement: Figure 2—source data 3. [file elife-84792-fig2-data3.zip › Figure2-source_data_3/Figure2-source_data3_actin.png]

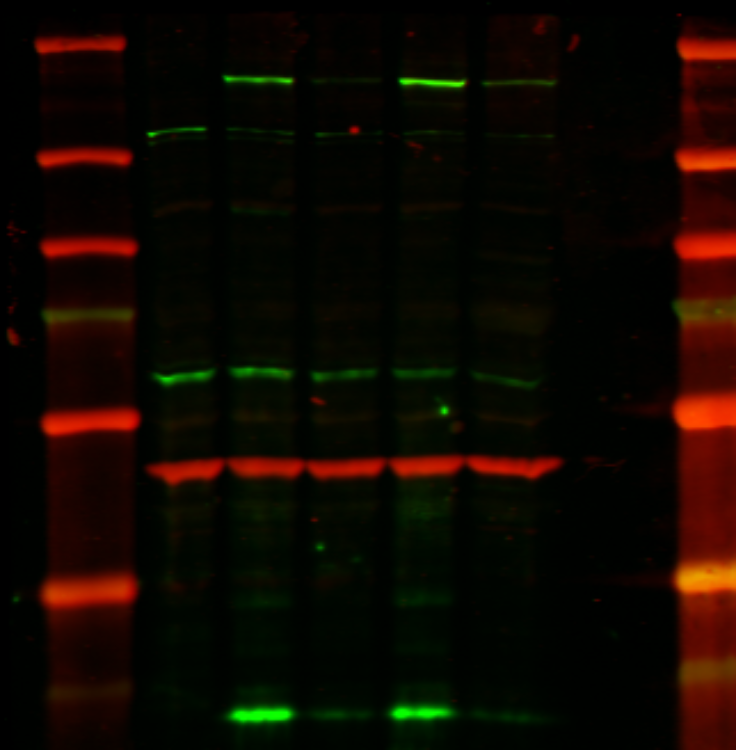

Supplement: Figure 2—source data 3. [file elife-84792-fig2-data3.zip › Figure2-source_data_3/Figure2-source_data3_V5 actin_overlay.png]

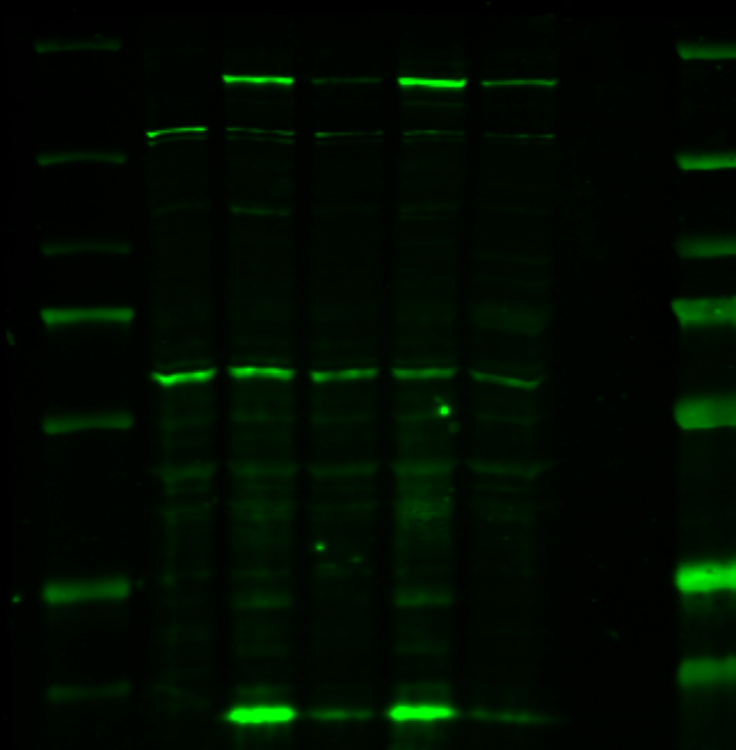

Supplement: Figure 2—source data 3. [file elife-84792-fig2-data3.zip › Figure2-source_data_3/Figure2-source_data3_V5.png]

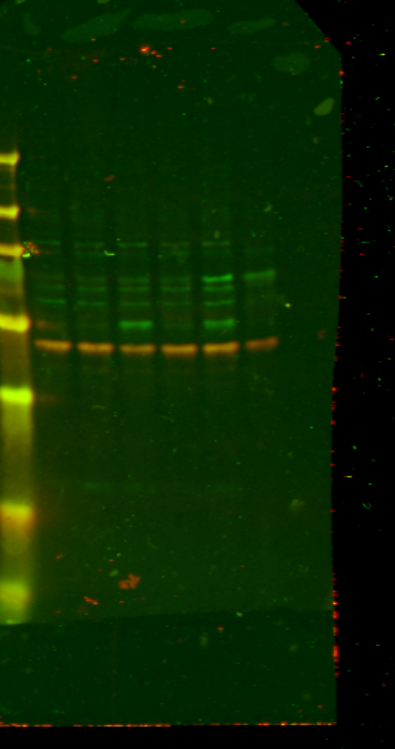

Supplement: Figure 2—figure supplement 2—source data 1. [file elife-84792-fig2-figsupp2-data1.zip › Figure2-figure_supplement2-source_data_1/Figure2-figure_supplement_2_source_data1_dsRed_green_actin_red.png]

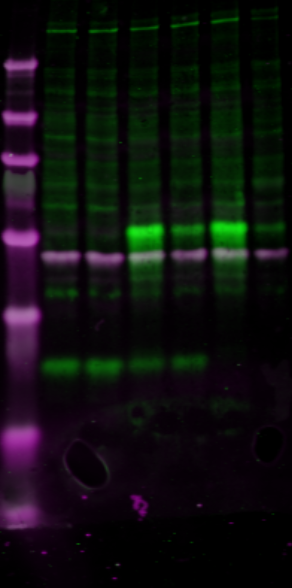

Supplement: Figure 2—figure supplement 2—source data 1. [file elife-84792-fig2-figsupp2-data1.zip › Figure2-figure_supplement2-source_data_1/Figure2-figure_supplement_2_source_data1_KRAS_green_Actin_magenta.tif]

Precision RNAi using synthetic shRNAmir target sites

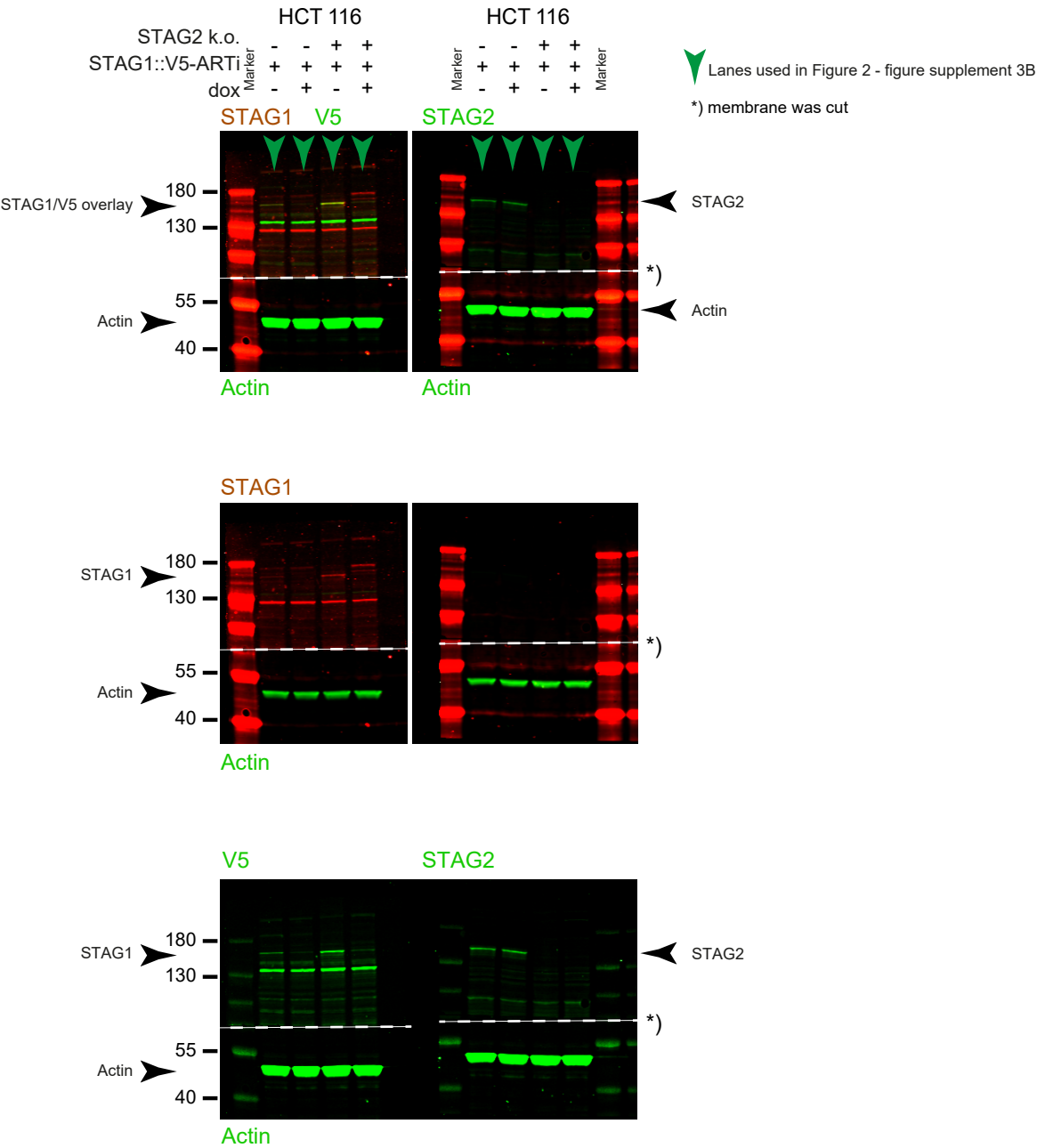

Supplement: Figure 2—figure supplement 3—source data 1. [file elife-84792-fig2-figsupp3-data1.zip › Figure2-figure_supplement3-source_data_1/Figure2_figure_supplement_3_source_data1_annotated.pdf]

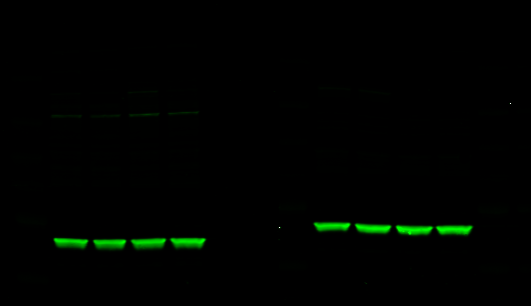

Supplement: Figure 2—figure supplement 3—source data 1. [file elife-84792-fig2-figsupp3-data1.zip › Figure2-figure_supplement3-source_data_1/Figure2-figure_supplement3-source_data_1_actin.tif]

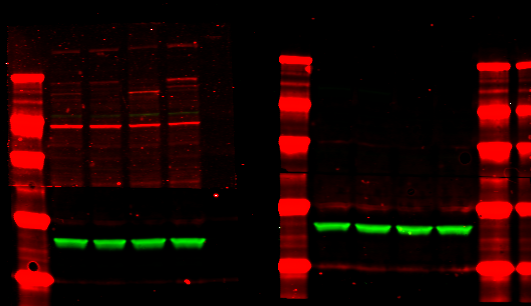

Supplement: Figure 2—figure supplement 3—source data 1. [file elife-84792-fig2-figsupp3-data1.zip › Figure2-figure_supplement3-source_data_1/Figure2-figure_supplement3-source_data_1_actin_stag1.tif]

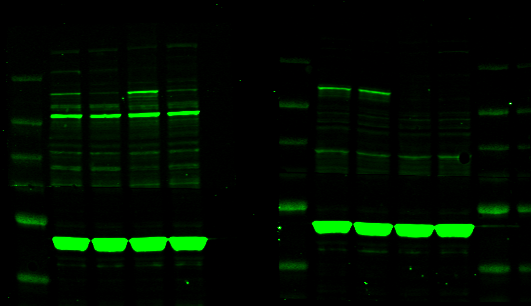

Supplement: Figure 2—figure supplement 3—source data 1. [file elife-84792-fig2-figsupp3-data1.zip › Figure2-figure_supplement3-source_data_1/Figure2-figure_supplement3-source_data_1_actin_v5_stag2.tif]

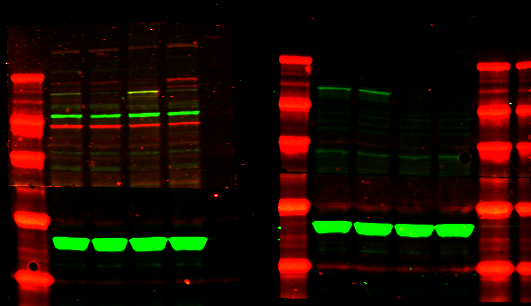

Supplement: Figure 2—figure supplement 3—source data 1. [file elife-84792-fig2-figsupp3-data1.zip › Figure2-figure_supplement3-source_data_1/Figure2-figure_supplement3-source_data_1_overlay.tif]
