# Supplementary material for: Precision RNAi using synthetic shRNAmir target sites
Source: eLife. 2023 Aug 8;12:RP84792. doi: 10.7554/eLife.84792 (PMC10409502; doi:10.7554/eLife.84792)
Supplement: Figure 2—figure supplement 2—source data 1. [file elife-84792-fig2-figsupp2-data1.zip › Figure2-figure_supplement2-source_data_1/Figure2_figure_supplement_2_source_data1_annotated.pdf]

Precision RNAi using synthetic shRNAmir target sites

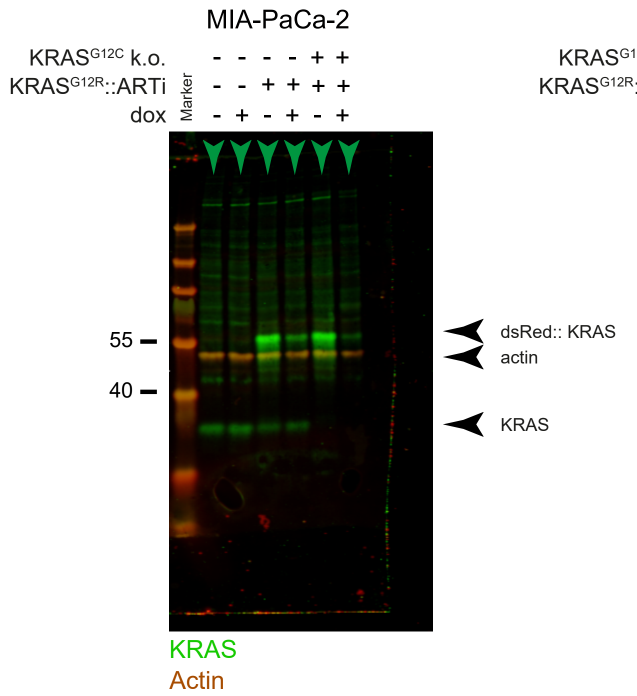

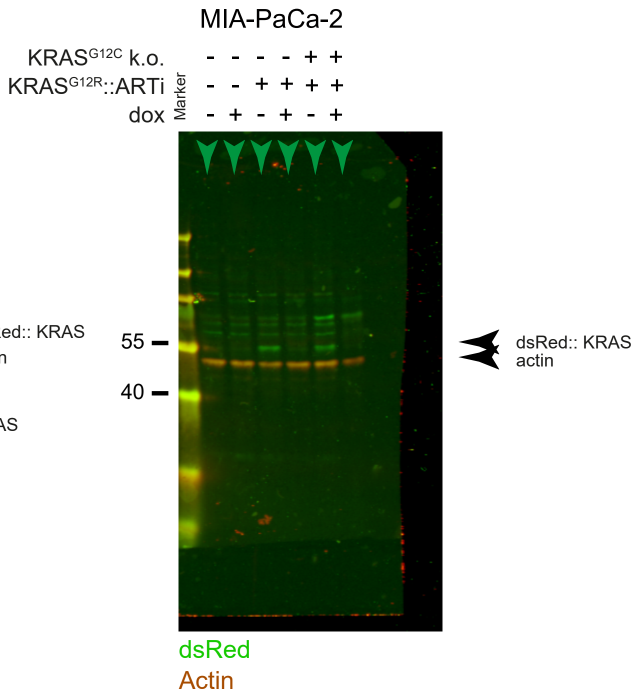

Lanes used in Figure 2F and Figure 2 - figure supplement 2D
